# Does Ad Hoc Coronary Intervention Reduce Radiation Exposure? – Analysis of 568 Patients

**DOI:** 10.5935/abc.20150110

**Published:** 2015-11

**Authors:** Márcio A. M. Truffa, Gustavo M.P. Alves, Fernando Bernardi, Antonio Esteves Filho, Expedito Ribeiro, Micheli Z. Galon, André Spadaro, Luiz J. Kajita, Raul Arrieta, Pedro A. Lemos

**Affiliations:** Instituto do Coração - Hospital das Clínicas - Faculdade de Medicina da Universidade de São Paulo (USP), São Paulo, SP − Brazil

**Keywords:** Angioplasty, Balloon, Coronary, Cardiac, Catheterization, Coronary Angiography, Fluoroscopy, Radiation Monitoring

## Abstract

**Background:**

Advantages and disadvantages of ad hoc percutaneous coronary intervention have been described. However little is known about the radiation exposure of that procedure as compared with the staged intervention.

**Objective:**

To compare the radiation dose of the ad hoc percutaneous coronary intervention with that of the staged procedure

**Methods:**

The dose-area product and total Kerma were measured, and the doses of the diagnostic and therapeutic procedures were added. In addition, total fluoroscopic time and number of acquisitions were evaluated.

**Results:**

A total of 568 consecutive patients were treated with ad hoc percutaneous coronary intervention (n = 320) or staged percutaneous coronary intervention (n = 248). On admission, the ad hoc group had less hypertension (74.1% vs 81.9%; p = 0.035), dyslipidemia (57.8% vs. 67.7%; p = 0.02) and three-vessel disease (38.8% vs. 50.4%; p = 0.015). The ad hoc group was exposed to significantly lower radiation doses, even after baseline characteristic adjustment between both groups. The ad hoc group was exposed to a total dose-area product of 119.7 ± 70.7 Gycm^2^, while the staged group, to 139.2 ± 75.3 Gycm^2^ (p < 0.001).

**Conclusion:**

Ad hoc percutaneous coronary intervention reduced radiation exposure as compared with diagnostic and therapeutic procedures performed at two separate times.

## Introduction

Interventional cardiological procedures, such as coronary angiography and percutaneous coronary intervention (PCI), are extremely important for diagnosis and treatment, have been increasingly used, but, so far, no alternative to radiation for their performance has been identified^1^.

The International Commission on Radiological Protection (ICRP) determines the risks of radiation exposure in fluoroscopy-guided procedures. Those risks are related to skin lesions (deterministic effects) and to an increase in the incidence of neoplasia (stochastic effects)^2,3^. The use of radiological imaging has increased, which, in association with the increase in life expectancy worldwide, is related to a considerable risk of cancer^4^.

A series of advantages and disadvantages of *ad hoc* PCI, such as that performed along with diagnostic catheterization, has been described. However, little is known about the radiation exposure of that procedure as compared to that of staged intervention, performed on a second occasion after the patient has undergone diagnostic catheterization.

The radiation doses of coronary angiography and interventional procedures, such as percutaneous coronary angioplasty, have been reported, mainly in complex procedures, the greatest doses being those of angioplasty^5-7^. However, no study has shown if the radiation doses of *ad hoc* and staged angioplasties differ.

This study was aimed at comparing the radiation exposure of patients undergoing two different PCI schemes: *ad hoc* and staged.

## Methods

### Study population

The present study included consecutive patients from one single center undergoing *ad hoc* (Group 1) and staged (Group 2) PCI between July 1^st^, 2012 and December 31, 2012. The procedures were performed at an academic institution, the Instituto do Coração (Incor) of the Hospital das Clínicas of the Medical School of the Universidade de São Paulo, by an attending physician accompanied by interventional cardiology trainees.

Demographic and procedural data were obtained from the electronic medical records of Incor and assessed in a historical prospective way. The following clinical variables were included: patient-related: sex, age, risk factors, clinical findings motivating catheterization, cardiac history and coronary anatomy; and procedure-related: number of lesions treated and stents implanted, and coronary artery territory approached. Both groups had clinically stable and unstable patients.

### Measures of radiation exposure

Radiation exposure was expressed as follows: Kerma (kinetic energy released per unit mass), which refers to the radiation beam delivered to the environment at a certain point; and ‘dose-area product’ (DAP), equivalent to the dose multiplied by the area irradiated. Kerma was quantified in Gy, and DAP, in Gycm^2^. We used the DAP because it bears a strong relationship with the dose effectively transmitted to the patient^6^. Such measures are integrated with the X-ray system and are available at the end of the procedure. In addition, fluoroscopic time and number of acquisitions were computed and compared. In the group of staged angioplasty, the radiation measurements of diagnostic coronary angiography were added to those of angioplasty.

The procedures took place at the catheterization laboratory of Incor, which has five rooms, four of which equipped with the Philips Allura Xper FD10 device, and one, with the Philips Allura Xper FD20 device. The acquisition field was 15- to 25-cm diagonal. The acquisition mode and number of frames varied between 15 and 30 frames/second.

### Statistical analysis

The Statistical Package for the Social Science (SPSS) software was used for statistical analysis. Continuous variables were expressed as mean and standard deviation, and categorical variables, as percentage. The groups were compared by using the non-parametric Mann-Whitney U test for continuous variables, and the chi-square or Fisher exact test for categorical variables. At the end, the dependent variable ‘total DAP’ underwent multiple regression with generalized linear models (GLM) for the dependent variable without normal distribution; gamma distribution, with logarithmic link function and backward selection method, was used. The initial model included the following predictive variables: procedure type (*ad hoc,* 1; staged, 0); age (years); sex (male, 1; female, 0); systemic arterial hypertension (yes, 1; no, 0); diabetes mellitus (yes, 1; no, 0); dyslipidemia (yes, 1; no, 0); smoking habit (yes, 1; no, 0); previous acute myocardial infarction (yes, 1; no, 0); previous PCI (yes, 1; no, 0); previous coronary artery bypass graft surgery (yes, 1; no, 0); previous congestive heart failure (yes, 1; no, 0); angiographic characteristic (single-, two-vessel: 0; three-vessel: 1); use of drug-eluting stent (yes, 1; no, 0); angioplasty of left main coronary artery (yes, 1; no, 0); angioplasty of anterior descending coronary artery (yes, 1; no, 0); angioplasty of right coronary artery (yes, 1; no, 0); angioplasty of circumflex coronary artery (yes, 1; no, 0); angioplasty of saphenous vein graft (yes, 1; no, 0); number of lesions (1, 0; 2 and 3, 1); number of stents (0, 1: 0; 2 to 5: 1); and total stent length (cm). The significance level adopted was α = 0.05.

## Result

This study included 568 patients, 320 of whom underwent *ad hoc* procedures (Group 1) and 248, staged procedures (Group 2). Table 1 shows the clinical and angiographic characteristics of the groups as means and percentages. The groups did not differ regarding risk factors for DAC, except for dyslipidemia and arterial hypertension, more common in Group 2.

**Table 1:** Clinical, angiographic and procedural characteristics

Total = 568					Ad hoc (n = 320)	Staged (n = 248)	p value
Age (years)					63.3 ± 12.00	63.9 ± 11.43	0.567*
Male sex (%)					70.3	68.5	0.718†
**Patient's characteristics (%)**							
				SAH	74.1	81.9	0.035†
				DM	37.5	41.9	0.324†
				DLP	57.8	67.7	0.020†
				Smoking	39.4	43.1	0.412†
				Previous AMI	30.0	30.6	0.941†
				Previous PCI	30.0	26.2	0.368†
				Previous CABG	17.8	19.8	0.630†
				Previous CHF	25.3	27.8	0.564†
**Angiographic characteristics (%)**							0.015‡
				Single-vessel	30.3	22.2	
				Two-vessel	30.9	27.4	
				Three-vessel	38.8	50.4	
**Procedural characteristics (%)**							
				DES^a^	20.5	32.7	0.001†
				LMC^b^	1.6	3.2	0.308†
				AD^b^	48.7	46.0	0.568†
				RC^b^	32.1	29.4	0.560†
				CX^b^	23.6	40.7	< 0.001†
				SVG^b^	6.9	4.0	0.196†
				Other vessels^b^	0.6	2.0	0.249§
				Lesions (number)^b^	1.22 ± 0.49	1.36 ± 0.54	< 0.001*
				Stents implanted (number)^b^	1.32 ± 0.83	1.65 ± 0.97	< 0.001*
				Total stent length (cm)^b^	26.01 ± 18.41	33.48 ± 22.82	< 0.001*

* Non-parametric Mann-Whitney U test;

† Yates correction for 2 x 2 tables;

‡ Pearson correlation;

a data from three patients of the ad hoc group missing;

b data from two patients of the ad hoc group missing;

§ Fisher exact test. Results presented as mean (standard deviation) or percentage.

Results presented as mean (standard deviation) or percentage. SAH: Systemic arterial hypertension; DM: Diabetes mellitus; DLP: Dyslipidemia; AMI: Acute myocardial infarction; PCI: Percutaneous coronary intervention; CABG: Coronary artery bypass graft surgery; CHF: Congestive heart failure; DES: Drug-eluting stent; LMC: Left main coronary artery; AD: Anterior descending coronary artery; RC: Right coronary artery; CX: Circumflex artery; SVG: Saphenous vein graft.

Group 1 patients more often had single- and two-vessel angiographic characteristics than Group 2 patients, in whom the three-vessel pattern predominated. Group 2 as compared to Group 1 had a higher number of lesions treated (1.22 ± 0.49 vs. 1.36 ± 0.54; p < 0.001), requiring a greater number of stents (1.32 ± 0.83 vs. 1.65 ± 0.97; p < 0.001) and longer stent length (26 ± 18.4 vs. 33.48 ± 22.8; p < 0.001) (Table 1).

**Figure 1 -:**
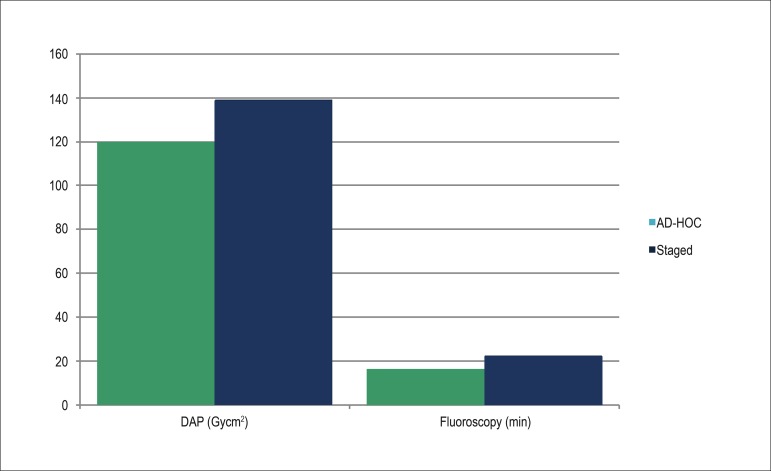
Radiological characteristics of the procedures (p < 0.01). DAP: dose-area product.

The comparison of the radiological characteristics between both groups is expressed as mean ± standard deviation in Table 2 and illustrated in Figure 1. Group 1 patients as compared to Group 2 patients underwent a smaller amount of radiation expressed in Kerma (Group 1: 3.4 ± 12.6 Gy; Group 2: 9.3 ± 60.8 Gy; p < 0.001) and DAP (Group 1: 119.7 ± 70.7 Gycm^2^; Group 2: 139.2 ± 75.3 Gycm^2^; p < 0.001), a shorter fluoroscopic time (Group 1: 16.5 ± 10.1 minutes; Group 2: 22.4 ± 14 minutes; p < 0.001), and a smaller number of acquisitions (Group 1: 26.3 ± 9.6; Group 2: 31.6 ± 10.9; p < 0.001).

**Table 2:** Radiological characteristics of the procedures

	Ad hoc (n = 320)	Staged (n = 248)	p value
Total Kerma (Gy)	3.4 ± 12.6	9.3 ± 60.8	< 0.001 *
Total DAP (Gycm^2^)	119.7 ± 70.7	139.2 ± 75.3	< 0.001 *
Total fluoroscopic time (min)	16.5 ± 10.1	22.4 ± 14.0	< 0.001 *
Total acquisition (number)	26.3 ± 9.6	31.6 ± 10.8	< 0.001 *
Kerma per lesion (Gy)	3.1 ± 12.7	8.6 ± 60.7	0.082
DAP per lesion (Gycm^2^)	106.3 ± 67.3	112.1 ± 68.9	0.145
Fluoroscopic time per lesion (min)	14.3 ± 9	17.7 ± 11.2	< 0.01
Acquisition per lesion (number)	23.2 ± 9.6	25.2 ± 10.2	< 0.01

* Non-parametric Mann-Whitney U test; DAP: Dose-area product.

Table 3 compares patients according to their number of lesions treated. Those having only one lesion treated were exposed to lower radiation doses, and those having two or more lesions treated showed a tendency towards lower doses.

**Table 3:** Radiological characteristics of the procedures, with stratification according to the number of lesions treated

	Ad hoc	Staged	p value
**One lesion**	**(n = 260)**	**(n = 165)**	
Total Kerma (Gy)	3.5 ± 14.0	11.8 ± 74.2	0.006*
Total DAP (Gycm2)	115.3 ± 69.5	130.7 ± 74.1	0.007*
Total fluoroscopic time (min)	15.3 ± 9.3	20.1 ± 12.1	< 0.001*
Total acquisition (number)	25.1 ± 9.5	29.0 ± 10.0	< 0.001*
**Two or more lesions**	**(n = 58)**	**(n = 83)**	
Total Kerma (Gy)	2.9 ± 1.3	4.3 ± 8.9	0.103*
Total DAP (Gycm^2^)	141.6 ± 72.5	156.1 ± 75.1	0.203*
Total fluoroscopic time (min)	22.1 ± 11.9	26.8 ±1 6.4	0.121*
Total acquisition (number)	31.8 ± 8.0	36.6 ± 10.8	0.008*

* Non-parametric Mann-Whitney U test; DAP: Dose-area product.

Table 4 shows the multiple regression analysis for radiation exposure. The predictors related to the increase in radiation exposure were the number of stents implanted (two or more) and the three-vessel pattern.

**Table 4:** Variables associated with the total dose-area product (Generalized Linear Model, with gamma distribution and logarithmic link function), n = 566

Variable	Coefficient	SE coef	p value
Number of stents (2-5)	0.301	0.049	< 0.001
Angiographic characteristic (three-vessel)	0.175	0.048	< 0.001
Constant	4.646	0.035	< 0.001

SE coef: Standard error of the estimated coeffiaent

## Discussion

The major finding of this study performed with consecutive patients undergoing angioplasty in one single center was hat those submitted to the *ad hoc* strategy as compared to those submitted to the staged strategy received a smaller amount of radiation (expressed in Kerma and DAP) and had a shorter fluoroscopic time and a smaller number of acquisitions.

The doses used were greater than those of previous studies^6,8^. Considering only the doses used in angioplasties, previous studies have reported mean DAP of 55 Gycm^2^ and 86.2 Gycm^2^, while, in this study, it was 119.7 Gycm^2^. In addition to the greater complexity of the lesions treated in this study, with more three-vessel patients in both groups, that finding might relate to the fact that the procedures were performed in one single academic institution, involving interventional cardiology trainees, as already reported^9,10^.

Regarding the angiographic characteristics, a larger number of three-vessel patients was observed in the staged procedure group. However, of the 320 Group 1 patients, 58 (18.1%) had two or more lesions treated, while of the 248 Group 2 patients, 83 (33.5%) had two or more lesions treated. Comparing both subgroups, a clear tendency towards a smaller dose of radiation is observed in Group 1. This shows that, although Group 2 had a more complex anatomy, it did not increase the radiation dose.

Regarding the characteristics of the procedure, Group 2 had more angioplasties of the circumflex artery (CX), and a greater number of lesions treated and of stents implanted. A study with 1,827 patients undergoing angioplasty has shown that the complexity of the lesion treated, angioplasty of the CX and number of lesions treated correlated with an increase in the radiation dose^11^. Another study involving 20,669 procedures has shown that the treatment of two or more lesions correlated with an increase in radiation exposure^12^. Thus, the greater number of angioplasties of the CX and of lesions approached in Group 2 may have increased the need for radiation observed in that group. However, when assessing the radiation dose used per lesion treated, Group 1 maintained its advantage regarding lower dose, expressed as the fluoroscopic time and number of acquisitions, with a clear tendency towards lower Kerma and DAP values per lesion treated in that group.

Assessing the subgroup of patients having only one lesion treated (260 Group 1 patients and 165 Group 2 patients), a significant difference was observed in the radiation dose expressed in total Kerma (p = 0.006), total DAP (p = 0.007), total fluoroscopic time (p < 0.001) and total number of acquisitions (p < 0.001), favoring Group 1.

Delewi et al. have reported that the increase in radiation exposure of patients undergoing angioplasty and coronary angiography related to the following: body mass index, history of coronary artery bypass graft surgery, number of lesions treated and of chronic total occlusion lesions^12^. In our study, the variables related to increased radiation exposure were the number of stents implanted and the three-vessel pattern. Therefore, one may assume that patients undergoing a staged procedure, with several and complex lesions to treat, especially those obese and having previous coronary artery bypass graft surgery, might require a high radiation dose and previous planning of the procedure, aimed at minimizing the physician’s and patient’s radiation exposure. In addition, it is worth considering the *ad hoc* procedure, mainly in the presence of other variables related to increased radiation dose.

### Study limitations

This historical prospective study was conducted at one single center with data collection from medical records.

## Conclusion

Ad hoc percutaneous coronary angioplasty, as compared to staged angioplasty, was associated with a significant reduction in patient’s radiation exposure even after adjusting for baseline differences between groups, with smaller DAP and Kerma, shorter fluoroscopic time and smaller number of acquisitions. Our findings suggest that lower radiation doses can be seen as a potential benefit of *ad hoc* angioplasty.
